# An Adaptive User Tracking Algorithm Using Irregular Data Frames for Passive Fingerprint Positioning

**DOI:** 10.3390/s22197124

**Published:** 2022-09-20

**Authors:** Donghyun Kim, Kyuho Son, Dongsoo Han

**Affiliations:** School of Computing, Korea Advanced Institute of Science and Technology, Daejeon 34141, Korea

**Keywords:** indoor positioning, passive fingerprinting, user tracking, adaptive algorithm, location based service

## Abstract

Wi-Fi fingerprinting is the most popular indoor positioning method today, representing received signal strength (RSS) values as vector-type fingerprints. Passive fingerprinting, unlike the active fingerprinting method, has the advantage of being able to track location without user participation by utilizing the signals that are naturally emitted from the user’s smartphone. However, since signals are generated depending on the user’s network usage patterns, there is a problem in that data are irregularly collected according to the patterns. Therefore, this paper proposes an adaptive algorithm that shows stable tracking performances for fingerprints generated at irregular time intervals. The accuracy and stability of the proposed tracking method were verified by experiments conducted in three scenarios. Through the proposed method, it is expected that the stability of indoor positioning and the quality of location-based services will improve.

## 1. Introduction

People spend more time indoors. Research on indoor positioning and tracking is being actively conducted. An indoor positioning system involves a set of components that determine the locations of users in a building or underground spaces. Unlike an outdoor environment, which mostly depends on GPS, an indoor positioning system needs a different type of signal or sensor because the exterior building walls block GPS signals. Although many researchers have proposed various signals, such as RFID, UWB, and Bluetooth, Wi-Fi-based positioning is the most practical method, given the widespread use of wireless access points (APs) and mobile devices that communicate via Wi-Fi signals.

In most cases, Wi-Fi-based positioning refers to Wi-Fi fingerprint-based positioning, which needs to construct an indoor signal database called a ‘radio map’. The key element of Wi-Fi-based positioning is the received signal strength (RSS) value, which can be calculated when Wi-Fi frames are captured. With multiple RSS values and corresponding transmitter IDs, the signal fingerprint is created in the form of a vector. Since RSS values are different by geographic location, Wi-Fi fingerprints collected at each point become features that distinguish every location of indoor spaces.

Generally, Wi-Fi fingerprint positioning systems can be classified as active or passive fingerprint positioning systems. In active fingerprint positioning, mobile devices of users actively collect the RSS values of wireless APs to identify their locations. The collection process is conducted on the user’s side. In contrast, in the passive fingerprint method, the collection process is exactly the opposite. RSS values emitted from mobile devices are collected by collection devices.

While the active fingerprint method is more common, the active fingerprint method has a few disadvantages compared to the passive method. First, active fingerprinting requires the aggressive participation of the user. In the active fingerprint method, the mobile device is in charge of collecting signal information. To do this, the user must download a specially designed application and allow permission for Wi-Fi scanning. Second, the active fingerprint method highly depends on the type of mobile device. Several operating systems, such as iOS, do not permit user applications, so the active fingerprint method cannot be performed on the devices. Third, the periodic scanning process consumes a lot of battery power. A scanning process is performed every time the user asks for a position.

To overcome these limitations, many researchers have recently started to examine the passive fingerprint method. The passive fingerprint method also uses Wi-Fi RSS data but the responsibility of data collection is not on users. Collection devices, called Wi-Fi monitors, opportunistically capture the Wi-Fi frames that are emitted from the mobile devices of users. This structure does not require a scanning process on the user’s smartphone and makes it possible to track more people without encouraging participation.

The final goal of indoor positioning is to specify the trajectory of users, also known as tracking. In tracking scenarios, sequential fingerprints should be utilized, different from positioning scenarios. Since the positioning algorithms handle all fingerprints independently, pattern matching usually comes to the core process of the algorithm. However, in tracking algorithms, the pattern of signals as well as the previous location of the target user should be utilized to obtain precise and stable results based on the probabilistic correlation with the current location.

The Viterbi algorithm [1] is a dynamic programming method based on the hidden Markov model (HMM). Each probability of state is calculated following the transition probabilities among states. This feature makes the Viterbi algorithm the golden rule for indoor tracking. Regarding each state, as each position, transition probabilities depend on environmental factors and user behaviors. Reasoning on the current position of the target can be effectively supported by the optimal trace from the model. Therefore, as with many precedent studies on indoor tracking algorithms, this work exploits the Viterbi algorithm.

However, the passive fingerprint method relies on the user to obtain RSS data. Unlike the active fingerprint method, Wi-Fi monitors wait until the Wi-Fi frames are detected. The data frame, which occupies most of the frames used in the passive fingerprint, occurs when using the actual network. As a result, the time intervals of the generated fingerprints based on the captured frames are irregular. When the smartphone uses many network resources, the time interval between fingerprints becomes shorter, and when it uses less, the interval becomes longer. Since most studies using HMM as a tracking algorithm do not consider cases with irregular time intervals, they fixed the probability parameters. They show quite high accuracies when the intervals are short or constant, but when the interval is longer or nonuniform, the performance is drastically degraded.

Therefore, this work proposes an adaptive user tracking algorithm, which shows a stable performance even when the time intervals between fingerprints are nonuniform. The transition probability of the HMM is dynamically set each time a new fingerprint is observed according to the time interval of the fingerprint. In addition, the fingerprint modification method is also proposed to complement fingerprints and filter fake fingerprints at the same time. Through experiments of the various scenarios, it was confirmed that the proposed methodology shows a stable and high-tracking performance compared to the original HMM and Viterbi algorithms.

## 2. Related Works

### 2.1. Active Fingerprinting

Wi-Fi fingerprint-based location and tracking have been actively researched for quite some time; most research studies are on the active fingerprinting method. In active fingerprinting, the user’s smartphone actively scans the nearby APs to make a fingerprint and radio map. RADAR [2] is known as the first study of indoor positioning based on the Wi-Fi fingerprint. The authors used the *k*-nearest neighbor (k-NN) algorithm to determine a user’s location within the building. After RADAR, many researchers applied k-NN to fingerprint-based positioning problems. Furthermore, Reference [3,4] proposed the methods using a probabilistic algorithm for positioning. It is computationally more expensive than the k-nn algorithm, but it is more accurate and stable. Among various types of tracking strategies, using the hidden Markov model (HMM) with the Viterbi algorithm is known to have high accuracy compared to other algorithms (because it comprehensively considers the user’s movements along with the RSS value).

The authors of [5,6] applied HMM to solve the tracking problem and achieved almost 90% accuracy when comparing the proximity results to the ground truth labeled for each site. They determined the probability parameters of HMM based on the fingerprint saved in the radio map. The authors of Reference [7] attempted to reflect the displacement of the user in HMM using inertial sensors on a smartphone.

However, since the active fingerprint method requires mobile devices to collect data, positioning is possible only for users who have installed a special application. Some mobile devices do not support the Wi-Fi scanning API, which is the core function of the app, so positioning is not possible even if the user wants it. In addition, the app for data collection consumes a lot of battery power, which is inconvenient for users.

### 2.2. Passive Fingerprinting

To jump over the hurdles of active fingerprinting, the passive fingerprinting method uses Wi-Fi frames generated by the user’s mobile device. Generally, two types of frames are emitted from mobile devices: the probe request frame and the data frame. The probe request frame is the frame sent by the client to scan the available WLAN network. According to [8], the probe request frames are transmitted at intervals of 10 to 15 s to search for nearby APs. In the case of the data frame, it is generated whenever the smartphone uses network resources.

Many studies have utilized probe request frames for indoor positioning. Reference [9] introduced a passive Wi-Fi tracking system called Probr, but they used a multilateration algorithm that degraded the performance in a complex indoor space due to the multi-path effect, the phenomenon of fluctuations in signal strength formed by an incoherent combination of signals coming from different directions through reflection or scattering with the direct signal [10]. Reference [9,11] also proposed their own method for the passive fingerprint positioning method, but they focused on detecting whether users passed certain rooms or zones; they implemented simple monitoring systems, such as counting visitors and crowd analyses. Reference [8,12] made a radio map and proposed a positioning method with the *k*-nn and weight-*k* naive Bayes algorithm, respectively. However, due to the long transmission period of the probe request frame, it took too long to collect enough RSS data to track the user. Reference [13] analyzed the social behaviors and patterns rather than the locations of users through data frames and probe request frames. Reference [14] used data frames to localization but not to track the users.

Reference [15] focused on a passive fingerprint positioning method with flow control signals, such as RTS/CTS and data frames accessing infra AP kernels. The research achieved 0.8 m/1.5 m of positioning errors by data frames in used/unused scenarios utilizing RSSI and CSI in an office environment. Utilizing CSI tends to be dependent on environmental factors, such as structures of the building and pedestrian behaviors. In addition, infra AP kernels can be hard to access. Despite these obstacles, the technique can be a good solution in well-controlled user scenarios because of its remarkable performance.

Even though there were numerous attempts to apply HMM and the Viterbi algorithm in the active fingerprint tracking method, there were fewer tries in the passive fingerprint. In [16], the authors utilized HMM to track unmodified smartphones using probe request frames from mobile devices due to insufficient probe request frames. Therefore, in this work, we propose an HMM-based user tracking algorithm in passive fingerprinting using both types of frames. The challenges of applying HMM and the Viterbi algorithm in passive fingerprint positioning using data frames are explained in the next chapter.

## 3. Challenges

**Figure 1:**
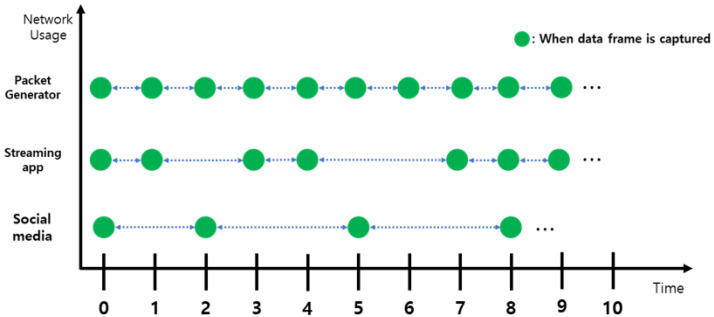
Time interval variations between frames according to the application.

In active fingerprinting methods, the user’s smartphone actively scans nearby APs at periodical times. However, in passive, the Wi-Fi monitor that is in charge of data collection waits until the frames arrive. Since data frames are generated when the smartphone is using actual network resources, the time intervals between frames become irregular. As shown in Figure 1, the data frame interval can be diverse according to the type of smartphone application. If the target user is watching a video through a streaming application, the smartphone will use a large number of network resources, which causes it to emit many data frames in a short time. In contrast, if social media or messaging apps are running on the smartphone, the amount of sending data will be relatively small than using a streaming application, such as YouTube.

Irregularly occurring data frames create successive fingerprints with random time intervals. Therefore, at some timestamps, there may not be enough frames to create the fingerprint, and it may be difficult to infer the next position based on the previous position because the time difference between the adjacent fingerprints is too large. As a result, stable tracking accuracy cannot be guaranteed as the time interval changes.

Moreover, the passive fingerprinting method uses multiple Wi-Fi monitors to capture the frames. Each monitor collects frames over a period and sends them to the server. Although all monitors have the same collection period, it is almost impossible to synchronize the collection start time. This can lead to a situation where two monitors report different timestamps of a data frame that was actually sent to the same specific timestamp. Therefore, when creating a fingerprint, RSS values of the same frame can be included in one fingerprint, even if the recorded timestamp values are different.

To overcome these problems, this paper suggests two ideas: the fingerprint modification process and the time interval adaptive tracking algorithm. Through the modification process, only valid fingerprints are obtained by filtering the fingerprints that have few RSS values. The time interval adaptive tracking algorithm receives the fingerprint sequence as well as a list of time differences from the previous fingerprint as input and reflects the time intervals to compute the trajectory of the user. The specific methods of the suggested ideas will be described in detail in Section 4.

## 4. Proposed Methods

In this section, the process of passive fingerprint-based tracking will be introduced. In Section 4.1, the overall tracking procedure will be described briefly and the details of each process will be explained throughout the rest of this section.

### 4.1. Overview

**Figure 2:**
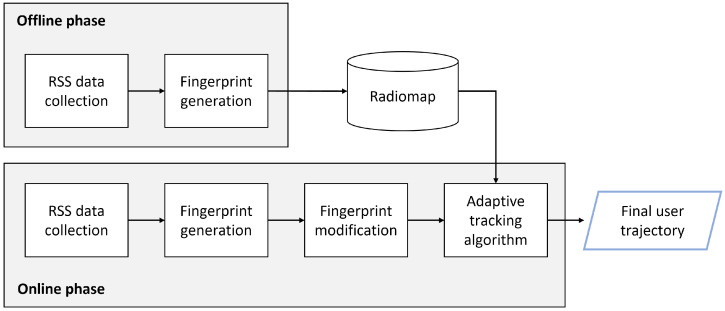
Overview of the user tracking process in the passive fingerprint.

Figure 2 shows the overall user tracking process in the proposed method. Before starting the process, Wi-Fi monitors are placed in an indoor space to collect RSS data from the users’ mobile devices. There is no rule of thumb for the positioning of the Wi-Fi monitors, but they should be evenly distributed throughout the entire space rather than concentrated on one side. Of course, the locations of monitors should be the same in the offline and online phases. Whenever deployed Wi-Fi monitors receive any frames from mobile devices, they send data containing the transmitter’s timestamp, MAC address, and RSS value to the server. The server then classifies the received data by the monitor ID and stores them in the database.

In the offline phase, the principal goal involves the construction of the radio map. The radio map constructor walks through the target space while carrying a smartphone. A packet generator app is run on the smartphone so that the Wi-Fi frames are emitted at 10∼50 ms of the time interval, which is short enough for the radio map construction without any data preprocessing. Since the signal strength varies with the distance and building structure, two distinct monitors have different RSS values on the frame. The frames from the smartphone are collected by the Wi-Fi monitors and sent to the server. After the constructor covers the entire target space, the server generates fingerprints by composing RSS values collected by all monitors concurrently. Frame data from different monitors are considered as sent from the same location if the timestamp is the same. The coordinates of the generated fingerprints are assigned along the path covered by the constructor. Finally, the radio map is constructed and stored in the database as a 〈key,value〉 structure where the key is the location coordinate and the value is the corresponding fingerprint.

The online phase is the stage where the actual user tracking is conducted. The data collection step is almost identical to the offline phase, except that the targets of the data collection (of RSS values) are users rather than the radio map constructor. The Wi-Fi monitors report the collected frames to the server and the server generates fingerprints of the target users. Unlike the offline phase, the time intervals of frames are longer and irregular in practical scenarios. In these situations, fingerprints that mostly consist of null values appear because of the report time differences among frame monitors. In this work, they are called ‘lacking fingerprints’. Therefore, a fingerprint modification method is needed to accurately estimate the trajectories of users by compressing ’lacking’ fingerprints into valid fingerprints, preserving valid RSS values. More details about the fingerprint modification process are explained in Section 4.4.

Although fingerprints are complemented by the modification process, the modified fingerprints are not ’time-evenly’ separated. As mentioned in Section 3, because of irregularly generated data frames, the frame interval adaptive algorithm is necessary for accurate and stable tracking results. The proposed adaptive algorithm utilizes HMM and the Viterbi algorithm. First, the algorithm searches for directly-reachable locations based on the time differences of the consecutive fingerprints and the previous location of the user. Then, it sets the transition probabilities based on whether the location is directly reachable or not. If the user can arrive within a given time, it assigns a high probability value, if not, it assigns a low probability value. This process is repeated *k* times, where *k* is the number of all online fingerprints. Emissions probabilities are also determined by the similarity of online fingerprints and all fingerprints saved in the radio map. Calculations using these probabilistic parameters provide a continuous location for mobile users. The offline phase process is explained in the next section.

### 4.2. Offline Phase

As mentioned before, all processes in the offline phase are for radio map construction. Until now, various radio map construction methods, such as point-by-point manual calibration (PMC), walking survey, and crowdsourcing-based have been studied. Each method has pros and cons, but in this work, the radio map was constructed by applying the walking survey method because it shows an appropriate performance at a reasonable time cost. Originally, it was invented in the active fingerprint method, but it can also be applied in a passive way.

In the walking survey method, the surveyor plans several routes that cover a whole indoor space. Then, the constructor walks along the planned survey routes while emitting Wi-Fi frames via the smartphone in his/her hand. The constructor records the start and end timestamps of each survey. Since the constructor knows the start point, endpoint, and time taken of the survey route, it is possible to assign the location of the RSS value based on the timestamp of the frame. In this work, the RSS value list sorted by the timestamp is sliced according to the number of discrete coordinates. For example, if the time required for one path is 60 s and the number of discrete coordinates in the path is 30, all collected RSS values are sliced in units of 2 s. By integrating the divided RSS values for each monitor, 30 fingerprints are created. From the start point of the survey route, each fingerprint is assigned to one coordinate. During the construction process, the constructor uses a high-rate packet generator application to make a dense radio map.

### 4.3. RSS Data Collection and Fingerprint Generation

**Figure 3:**
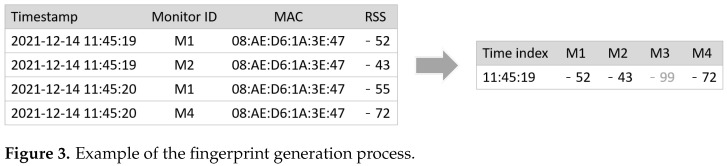
Example of the fingerprint generation process.

In passive fingerprinting, fingerprints are generated by RSS data collected by Wi-Fi monitors. Deployed Wi-Fi monitors capture frames in the air and then extract the MAC address of the transmitter and RSS value of each frame. After that, they periodically send the data in the form of {timestamp,MAC,RSS} with their own monitor ID. If *m* mobile devices send frames simultaneously, the format is {timestamp,$[\left(MAC_{1},RSS_{1}\right),$$(MAC_{2},RSS_{2}),$⋯,$\left(MAC_{m},RSS_{m})]\right\}$. The collection period of the Wi-Fi monitor is set to 1.4 s, validated by [17], and all Wi-Fi monitors synchronize the time by the network time protocol (NTP). Whenever the server receives RSS data from a monitor, it stores it in a database. The table on the left side of Figure 3 represents the database.

Unlike the offline phase, fingerprinting in the online phase requires a different approach. Since the distance of the user trace cannot be known in the online phase, all RSS values collected within a certain time window are reflected to make a fingerprint of the user.

Because the collection period of the Wi-Fi monitors is 1.4, some RSS values may be missing if the period is less than this. Therefore, in this work, the fingerprint generation period was set to 2 s. If multiple RSS values were presented within 2 s, the maximum RSS value of each monitor was selected. In case no values were collected during the period, the monitor’s RSS value was set to −99, which is theoretically the lowest value. Figure 3 shows an example of fingerprint generation, where *o* represents the online fingerprint. Please notice that −52 was selected for the RSS value of M1 because it is larger than −55 (captured a second later), and −99 was assigned for M3 because no RSS values were captured during the 2 s.

### 4.4. Fingerprint Modification

Even the fingerprint was generated through the process in Section 4.3; fingerprint filtering and complement processes are needed. In the passive fingerprint method, fingerprints are generated depending on whether the smartphone transmits frames. Whenever the smartphone is not emitting frames, fingerprints cannot be made. In other words, there may be a fingerprint in which all RSS values of Wi-Fi monitors are −99. To estimate the exact trajectory of the user, such fingerprints should be eliminated. The simplest filtering method involves ignoring the fingerprints where the −99 values are higher than the threshold; however, this method has disadvantages in that it can erase usable RSS values.

**Figure 4:**
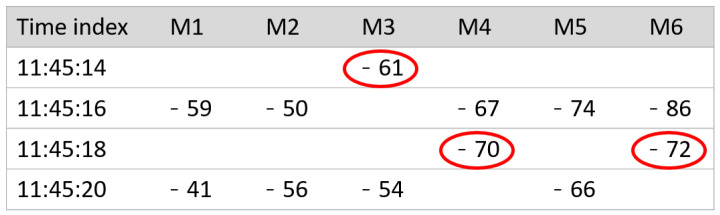
Example of the generated online phase fingerprint.

Figure 4 shows an example of online phase fingerprints. Each row represents a single fingerprint; the column name ‘Time index’ means the seconds after the user is first detected. The blank cell indicates the null (−99) RSS value, which is the default value when frames are not captured. In the figure, the RSS values with red circles can be exploited when merged with the previous or next fingerprint.

In the case of the −61 value of M3 at 11:45:14, it is obvious that the monitor M3 captured the frame a bit earlier because the RSS value of the next time index is empty. Therefore, the fingerprint of 11:45:14 should be merged into that of 11:45:16, reallocating the −61 value to the blank slot and M3 in 11:45:16. Other red circled values, −70 of M4 and −71 of M6 in 11:45:18, are similar cases. In this way, a fingerprint modification algorithm is proposed. The modification process compresses fingerprints by merging adjacent fingerprints.

Algorithm 1 shows the process of merging adjacent fingerprints. In lines 4∼9, scanning each element of observations *o*1 and *o*2, the value in *o*2 is adopted to the *merged* result, where the value in *o*1 is invalid. In lines 12∼17, the time index of the *merged* result is selected between *t*1 and *t*2, by contributions of *o*1 and *o*2.
**Algorithm 1:** Merge Observations
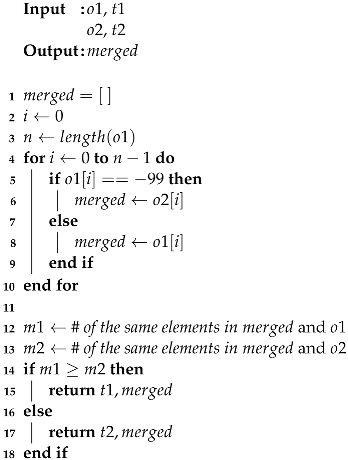


Algorithm 2 shows the main logic and process of fingerprint modification while Algorithm 1 concerns the mergeObservs function in Algorithm 2. There are three inputs for the fingerprint modification algorithm: *O*, *T*, and $N_{f}$. *O* is the array of online fingerprints sorted by the time index, *T* is the time index array of *O*. In the online fingerprint generation step, the generation period was set to 2 s, *T* is composed of multiples of 2, and the largest element is 2×(length(O)−1). $N_{f}$ is the threshold value for fingerprint filtering. If the number of −99 values in a certain fingerprint is greater or equal to $N_{f}$, then that fingerprint will not be used for tracking.

All fingerprints were checked to make sure they were meaningful fingerprints. In other words, we compared the number of −99 values of the fingerprints with $N_{f}$ (line 5 to 8). If there were enough valid RSS values, except for the first and the last fingerprint in *O*, every fingerprint merged with the previous and next fingerprint (*left*, *right*) through the mergeObservs function (line 11, 12). The mergeObservs function not only exchanged the null value with the valid value but also selected the proper time index of the merged fingerprints.

The mergeObservs function set the time index of the merged fingerprints by selecting the time index of one of the two given input fingerprints, which was more similar to the merged fingerprint. After merging, it picked which of the two merged fingerprints was well merged by counting null RSS values and appended to the *flitered* list. Since the first and last fingerprint could not merge with the next and previous fingerprints, respectively, only one merged fingerprint was appended.

Unlike the simple filtering strategy that eliminated all lacking fingerprints, the proposed method still provides a chance for RSS values with lacking fingerprints. Although a fingerprint is ignored due to the many null RSS values, some meaningful RSS values may exist. The proposed modification method extracts these valid RSS values while removing the lacking fingerprint itself. In this method, fingerprints with many null values were skipped, but the neighbor fingerprints referred to skipped fingerprints. After the modification process, only meaningful fingerprints remained, which were complemented with RSS values of adjacent fingerprints.
**Algorithm 2:** Fingerprint modification.
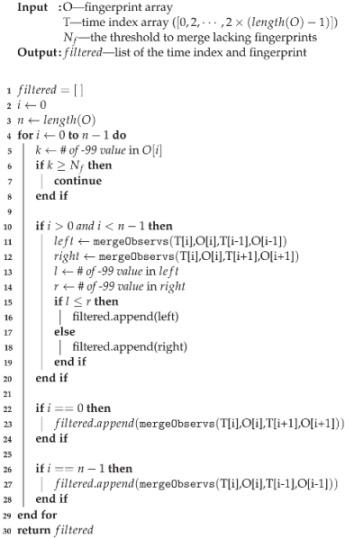


**Figure 5:**
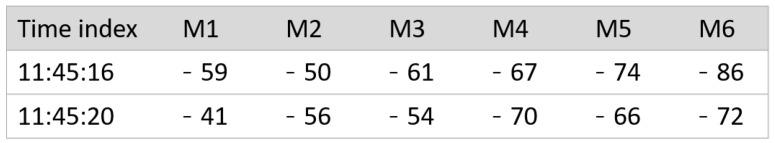
The fingerprints after the modification process.

Figure 5 shows the modified version of fingerprints in Figure 4. As expected, the valid RSS values in lacking fingerprints ($o_{1}4$ and $o_{1}6$) were combined with the next time index fingerprints. However, $o_{16}$ and $o_{18}$ were removed from fingerprint list because they had too many null RSS values. Through the modification process, reasonable fingerprints were obtained but the time intervals between fingerprints became larger than the time window of 2 s. In a general case, the time intervals between fingerprints can be diverse and irregular after the modification process. To track the user with irregular fingerprints, an adaptive algorithm is required and it will be explained in the next section.

### 4.5. Time Interval Adaptive Tracking Algorithm

When fingerprints are generated and modified in the online phase, they are input into a tracking algorithm to analyze the trajectory of the mobile user. The Viterbi algorithm, the most famous tracking algorithm, considers the fingerprint pattern as well as the relations between adjacent fingerprints. More precisely, it refers to the user’s previous location when determining the current location because the user cannot move far within a limited time. The HMM models the fingerprint pattern similarity, checking the process to emission probability and the geographical relationship analysis with the previous location to transition probability. Based on these characteristics, the Viterbi algorithm showed excellent performance in the indoor tracking field.

#### 4.5.1. Irregular Fingerprints in HMM

In Viterbi algorithms, there is a basic assumption that all observations are acquired at equal time intervals. In most cases of indoor tracking situations, the data are collected through certain objects actively and regularly. Therefore, the probability parameters of HMM are fixed at the beginning of tracking and are never changed during the tracking process.

However, in the passive fingerprint, the regularity of timestamps between consecutive fingerprints is not ensured. Even though the probe request messages are known to be generated periodically, data frames are not. The time intervals of data frames vary depending on the type of application that runs on the user’s smartphone and the way to use applications.

**Figure 6:**
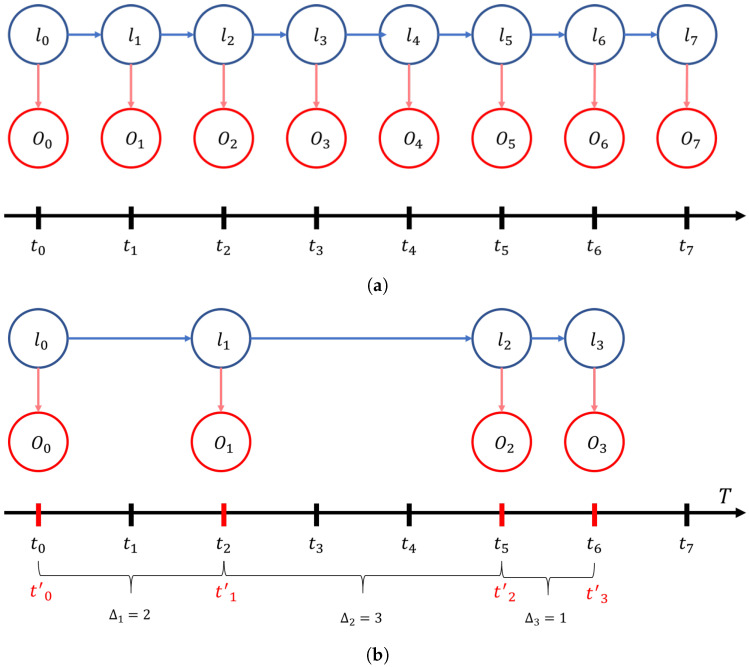
HMM when observation intervals are regular (**a**) and irregular (**b**).

Figure 6 shows the HMM modeling when the time intervals between fingerprints are regular and irregular. The blue arrows indicate the transition probabilities and the red arrows indicate the emission probabilities. In both regular and irregular cases, the lengths of the red arrows are not changed but the lengths of the blue arrows are random. It seems as if the transition probabilities should have different values depending on the time interval between observations.

Transition probability concerns the probability that a user will move from one location to another; it is obvious that the location near the base location has higher transition probability values than a location far from it. However, the actual meaning of the word ‘near’ is about direct reachability. A long time interval increases the number of nearby locations because the user has more time to travel further than a short time interval.

Moreover, it is clear that these directly-reachable locations have high probabilities and must have high transition probabilities, while not-directly-reachable locations have low probabilities. In other words, according to the time interval, the transition probability value from one location to another should be flexible. Therefore, the following section describes the strategy for dynamically setting the transition probability.

#### 4.5.2. Adaptive Transition Probability

The transition probability in HMM is related to the moving distance of the target user. As mentioned above, since there are not many cases where the observation time interval is irregular, most of the papers used a fixed transition probability according to the distance from the reference location. On the other hand, in calculating the transition probabilities in this work, the time interval Δt is differently applied depending on the time-lapse of every discrete neighboring fingerprint. As a result, the transition probability is newly computed for every location whenever a new fingerprint is observed.

The key idea is to nominate more candidates for directly-reachable locations as time intervals become longer. Since distance is the product of time and speed, if there is no significant change in the user’s speed, time is the only factor that affects the user’s moving distance. Unlike the outdoor environment, there is no event that drastically changes the speed of human movement, and the distance traveled in the indoor space is proportional to time. Therefore, in this work, a new distance function is defined that not only considers the physical distance between two different points but also utilizes time as a parameter.

Euclidean distance is a popular formula to calculate physical distance. However, in a complicated indoor space, the Euclidean distance can generate errors when computing the distances of two different locations along a corner. Therefore, the Manhattan distance is used for the distance measurement. The Manhattan distance formula is given when *D* is the dimension of the input:

(1) $$\begin{array}{cc} d_{1}\left(p,q\right) & =||p-{q||}_{1}\\ \; & =\sum_{k=1}^{D}\left|p_{k}-q_{k}\right| \end{array}$$

Therefore, the new distance function is defined below

(2) $$dist\left(l_{i},l_{j},\Delta t\right)=\left\{\begin{array}{ccc} \; & m_{i}\; & d_{1}\left(l_{i},l_{j}\right)\leq v\Delta t\\ \; & d_{1}\left(l_{i},l_{j}\right)\; & d_{1}\left(l_{i},l_{j}\right)>v\Delta t \end{array}\right\}$$

where,

(3) $$m_{i}=\min_{1\leq k\leq N}d_{1}\left(l_{i},l_{k}\right)\;\;\left(k\neq i\right)$$

In the proposed distance function, if the $d_{1}\left(l_{i},l_{j}\right)$ is less or equal to vΔt, this is considered a directly reachable location. Then $m_{i}$, which is the minimum Manhattan distance of $l_{i}$ to $l_{j}$, is selected as the value of the function. Here, *v* is the maximum speed of the target user that is empirically set as 0.5 m/s. If $d_{1}\left(l_{i},l_{j}\right)$ is greater than vΔt, this is considered a not directly reachable location. In this case, the Manhattan distance value itself is selected as the value of the function. The conditional formula makes every transition, considered as directly-reachable, according to the empirical maximum speed having the same probability. The distance function provides robustness of the speed changes of the target user, under the maximum speed of 0.5 m/s, with this feature. We call this ’partial robustness’ on the user speed.

The principal rule of probability is that the sum of all probabilities must be 1 so the summation of the transition probability must also be 1. In other words,

(4) $$\sum_{j=1}^{N}a_{i,j}=1$$

where $a_{i,j}$ is the transition probability from $l_{i}$ to $l_{j}$ and *N* is the number of discrete indoor coordinates. Therefore, although the proposed distance function provides relative importance to locations, the importance value should be standardized from 0 to 1 while the sum of the value is 1. To accomplish this goal, the softmax function is used. The softmax function is a famous activation function used in the deep learning field, especially in multi-class classification. The function transforms the value of the final output layer into a percentage of the total output layer value. Equation (5) shows the expression of the softmax function.

(5) $$softmax\left(z_{i}\right)=\frac{\exp(z_{i})}{\sum_{j=1}^{n}\exp\left(z_{j}\right)}$$

Finally, the proposed adaptive transition probability consists of the distance function in Equation (2) and the softmax function in Equation (5). More precisely, it is in the form of the softmax function with the inverse of the distance function as an input. The reason why the inverse of the distance function value is the input is that the transition probability should have a higher value when the distance is short. Therefore, the transition probability is defined as below.

(6) $$\begin{array}{cc} a_{l_{i},l_{j}\left(\Delta t\right)}\; & =softmax(dist{\left(l_{i},l_{j},\Delta t\right)}^{-1})\\ \; & =\frac{\exp\left(\frac{1}{dist(l_{i},l_{j},\Delta t)}\right)}{\sum_{k=1}^{N}\exp\left(\frac{1}{dist(l_{i},l_{k},\Delta t)}\right)} \end{array}$$

**Figure 7:**
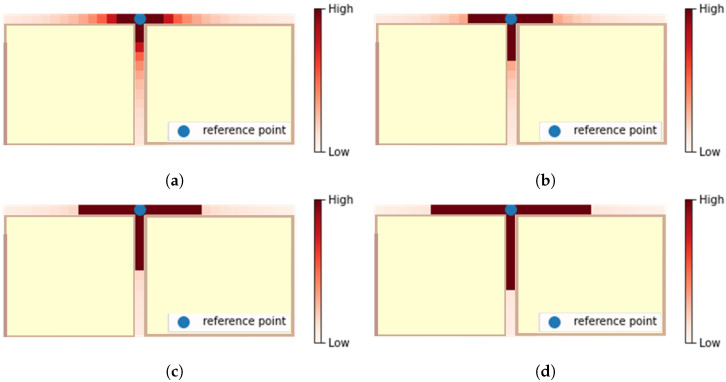
Heatmap of the proposed transition probability. (**a**) Δt=2, (**b**) Δt=4, (**c**) Δt=6, (**d**) Δt=8.

Figure 7 shows the heatmap of the proposed transition probability as Δt increases. The interior space consists of two corridors and one intersection. The blue point is the reference point $l_{i}$, and the areas painted in bright yellow are indoor spaces that cannot be occupied by the user due to the structure of the building. Positions are expressed in red as they have high transition probability values. As can be seen in the figure, as Δt increases, the number of positions expressed in red increases.

#### 4.5.3. Emission Probability

The emission probability is the probability that a particular fingerprint will be observed at that location. It can be expressed as $P(o_{t}|l_{i})$ or $b_{i}\left(o_{t}\right)$. Since the radio map is constructed in the offline phase, the emission probability could be induced by a similarity between the observed online fingerprint and the fingerprint saved in the radio map. The Manhattan distance is also used to calculate the similarity between the two fingerprints, as follows where *D* is the dimension of the fingerprint and $fp_{i}$ is the fingerprint saved in the radio map of location $l_{i}$. The denominator of the similarity function is the Manhattan distance value plus one. Here, ’one’ is to prevent in case the denominator becomes zero. In short, the similarity function is the reciprocal of the Manhattan distance mean between two fingerprints where 1 is added in the denominator.

(7) $$\begin{array}{c} similarity\left(o_{k},fp_{i}\right)=\frac{D}{1+d_{1}\left(o_{k},fp_{i}\right)} \end{array}$$

Moreover, let the softmax function used to ensure the sum of the emission probability be 1.

(8) $$\begin{array}{cc} b_{i}\left(o_{k}\right) & =softmax(similarity\left(o_{k},fp_{i}\right))\\ \; & =\frac{\exp\left(\frac{D}{1+d_{1}\left(o_{k},fp_{i}\right)}\right)}{\sum_{t=1}^{T}\exp\left(\frac{D}{1+d_{1}\left(o_{t},fp_{i}\right)}\right)} \end{array}$$

#### 4.5.4. Adaptive Viterbi Algorithm

Algorithm 3 shows the whole process of the proposed adaptive tracking algorithm. As input parameters, *S*, *O*, *A*, and *B* are given. *S* is the set of locations stored in the radio map, *O* is the list of collected and modified online fingerprints. *A* is the transition probability expressed in matrix form, and *B* is the emission probability matrix.
**Algorithm 3:** Time adaptive Viterbi
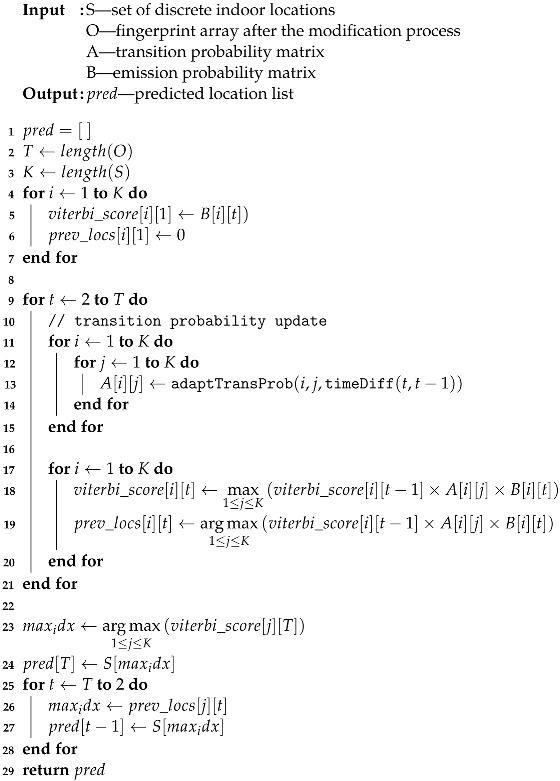


The mechanism of the algorithm is based on the Viterbi algorithm. Reference [6] shows a good example of utilizing the Viterbi algorithm in indoor tracking. Except for several newly defined factors in adaptive transition probability and emission probability, most parts follow the HMM/HsMM-based Viterbi algorithm in [6]. The proposed adaptive method, different from the existing Viterbi algorithm, which uses a predetermined static transition probability, updates transition probability through the adaptTransProb for each time; adaptTransProb is explained in (6), which uses the time difference Δt between the adjacent fingerprints to obtain the distances of two different locations. Here, the variable *t* in the algorithm indicates the index of the fingerprint list *O*. Therefore, to know the exact time difference between the adjacent fingerprints Δt, the timeDiff function is used.

The majority of the Viterbi algorithm calculates the probability by multiplying probability parameters and marks the previous location that had the highest probability. Finally, the most probable user trajectory is estimated through a backtracking process.

## 5. Results

### 5.1. Experiment Environment

**Figure 8:**
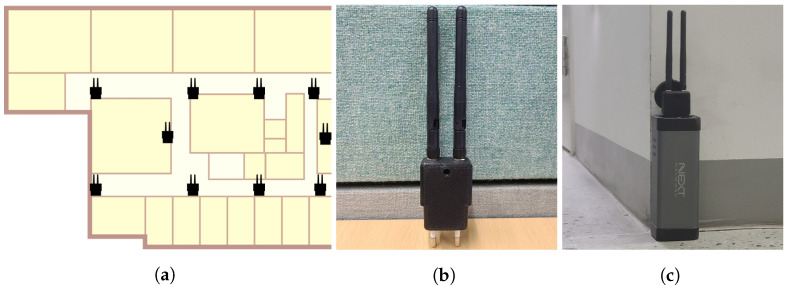
Environment settings. (**a**) The floor plan. (**b**) Wi-Fi monitor. (**c**) Deployment.

Figure 8a shows the floor plan of the indoor experiment space and the locations of Wi-Fi monitors. The experiment was conducted at the KAIST N1 building, we used half of the seventh floor. The experiment environment has multiple laboratories, professor’s offices, and seminar rooms. The experimental space is 30×13 m in size and has a corridor structure with a total of four different aisles and four three-way intersections. There are a total of 82 discrete indoor location coordinates divided into 1 m units. A total of 10 Wi-Fi monitors were deployed at intervals of 5~10 m apart to capture frames emitted from mobile devices and every monitor was connected to Wi-Fi to send the captured frame data. Before conducting the experiment on tracking, the radio map of the given space was constructed via the walking survey method, as explained in Section 4.2.

The appearances of the Wi-Fi monitors are shown in Figure 8a. The size of the monitor was 3.7×7×2.4 cm and it consisted of antennas, a body, and a plug. The antennas supported detecting frames and connected to Wi-Fi. The main body had two chips, ESP32 and ESP8266, which are widely used in the IoT field. ESP8266 is the chip responsible for frame capturing and data extraction. It captures frames emitted by the user’s mobile device with information about sending the device’s MAC address and the RSS value of the frame. The ESP32 sends the extracted data and a timestamp when it is captured. Since ESP32 sends the frame data to the server, it must know the IP address and port number of the server in advance. Moreover, ESP32 must connect to the network. Therefore, the system administrator must enter the target server’s IP address and port number into the ESP32 before deployment, and set up which network to connect to.

As shown in Figure 8b, when placing a monitor indoors, an AC output power bank was used to continuously supply power to the monitors. Each monitor was connected to one power bank and placed in the planned location. By doing this, the Wi-Fi monitor could be placed anywhere without worrying about electric outlets. In addition, it looked neater because the power strips were not required.

**Figure 9:**
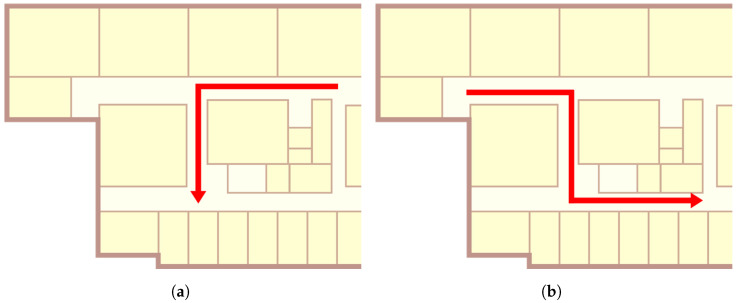
Test tracking routes. (**a**) Route 1, (**b**) Route 2.

Figure 9 shows the two test paths considered as the target user walked. Route 1 started from the upper right point of the floor map and made a left turn at the first intersection. Route 2, which was more complicated, started from the upper left side of the map and made a left turn and right turn at each crossroad. The route lengths were 29 and 39 m, respectively, and there was the assumption that the target user maintained the walking speed.

To experiment in a realistic situation, three different smartphone applications were run on the target user’s mobile device when the user traveled over the routes. The three applications were the packet generator, YouTube, and Instagram (social media service) in the order of the shortest data frame period. Therefore, a total of six experiments were conducted using three different applications per route.

### 5.2. Experiment Result

To compare the accuracy and stability of the tracking algorithms according to the time intervals of the data frames being changed, the quantitative value mean frame interval (MFI) was used. It was the value of the average time interval between frames. A smaller MFI value means that the target user’s smart data frames were generated in a shorter period, and more data frames were detected. Therefore, applications with small MFI values use more network resources than applications with large MFI values. However, even if the same application is used, the MFI value may change because the usage pattern is not exactly the same.

When $N_{M}$ is the number of Wi-Fi monitors, the MFI calculation process is as follows:

1. Create an array ($time\_list_{m}$) to store the timestamps of the RSS values collected in the Wi-Fi monitor $M_{m}$.
2. Create a frame interval array ($FI\_list_{m}$) to save the time difference between adjacent elements in $time\_list_{m}$.
3. Calculate the average of $FI\_list_{m}$: $avg(FI\_list_{m})$.
4. Repeat the above process for all Wi-Fi monitors ($M_{1},\ldots ,M_{N_{M}}$).
5. Find the minimum average of the frame interval array:
  $$\min_{1\leq m\leq N_{M}}\frac{\sum_{i=1}^{len(FI\_list_{m})}FI\_list_{m}\left[i\right]}{len(FI\_list_{m})}$$

The element in $time\_list_{m}$ is the timestamp when the monitor detects a Wi-Fi frame, so if the user is away from any Wi-Fi monitor, the monitor will drop the Wi-Fi frames not because the mobile device is not emitting frames, but because the frames cannot be reached. In this case, the MFI of the monitor might have a larger value than the actual MFI value. Therefore, to avoid this situation and obtain accurate MFI values, the minimum value of the average frame interval value for all Wi-Fi monitors is the final MFI of the experiment.

**Figure 10:**
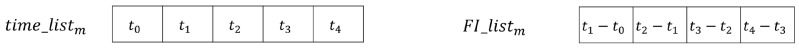
Step 2 in the MFI calculation process.

Figure 10 shows the second stage of the MFI calculation process when there are five elements in $time\_list_{m}$. Since the $FI\_list_{m}$ has a difference of two adjacent elements in $time\_list_{m}$ as the element, its size is one less than $time\_list_{m}$.

Table 1 shows the MFI value for each experiment. The packet generator application causes the smartphone to transmit countless dummy data frames in very short cycles. Because of this, the packet generator application has 1.54 and 1.42 as MFI values, which are almost the same as the collection period of Wi-Fi monitors. YouTube is a famous video streaming service. During the experiment with YouTube, the user watched 4*k* video and traveled Routes 1 and 2. Naver is a famous Korean website to watch the news or search for information; the experimenter watched the news with comments on it during the experiment. Instagram is a social media application that only allows images in the feed. In the experiment, the user viewed recommended posts and post comments.

Therefore, in the experiments, the user walked Route 1 and Route 2 while running four applications on the smartphone. Then the tracking results according to each scenario (that is, the results according to the changes in the MFI values) were analyzed.

#### 5.2.1. Route 1 Experiment

Tracking results can be compared through images or animations, but for a more accurate comparison, a method of numerically representing tracking accuracy was used. Assuming that the user maintained speed during the experiment, the length of the route and the time it took to move the experimental route determined the user’s speed. Based on this, given a timestamp of the fingerprint, we can calculate that the actual location of the user was the distance traveled during that time from the start of the experiment. Thus, the distance error of the tracking algorithm may be obtained by comparing the actual location and the predicted location.

**Figure 11:**
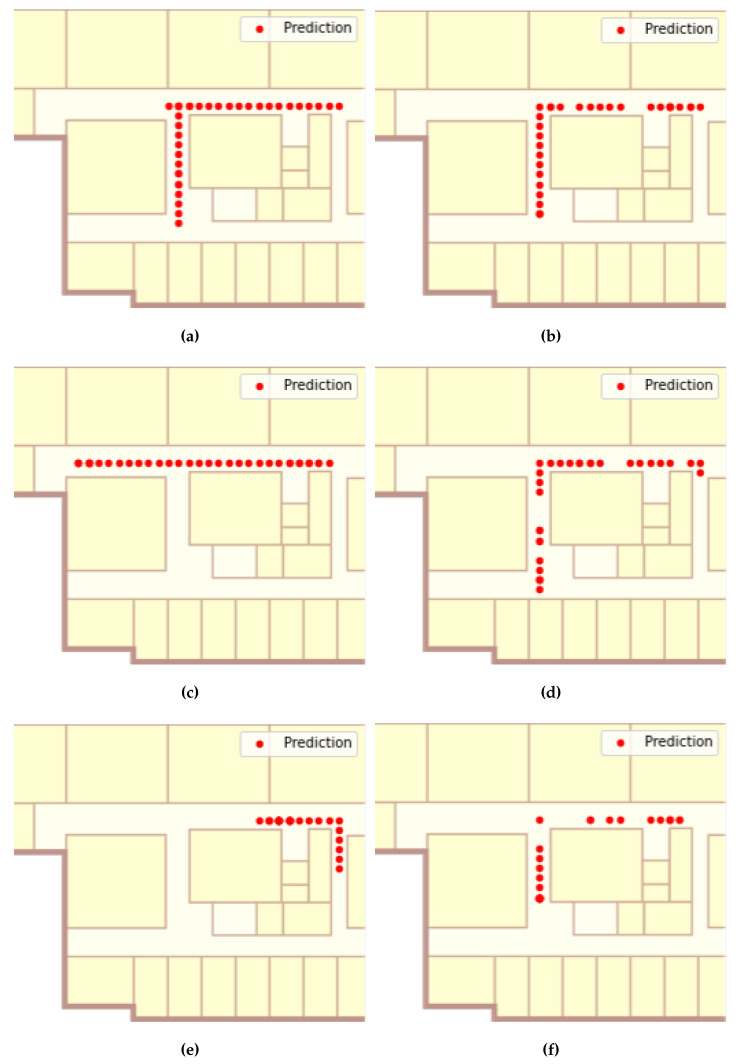
Tracking results on Route 1. (**a**) Viterbi to packet generator, (**b**) proposed method to packet generator, (**c**) Viterbi to YouTube, (**d**) proposed method to YouTube, (**e**) Viterbi to Instagram, (**f**) the proposed method to Instagram.

Figure 11 shows the tracking results of Route 1 while Table 2 indicates the distance error of the Viterbi algorithm and the proposed method. To filter out the cases where no RSS value was sent, the Viterbi algorithm removed fingerprints, where all values of the fingerprints were −99, and the proposed method performed the fingerprint modification process.

The proposed method, except for the packet generator scenario, shows better performance, and the accuracy difference is negligible even in the case of the packet generator. In the YouTube and Instagram scenarios, the Viterbi algorithm failed to follow the ground truth path of Route 1 (Figure 9a). In the YouTube scenario, the Viterbi algorithm estimated that the user was walking in a straight line, but in reality, the user turned left at the corner. In the Instagram scenario, with the worst tracking results, Viterbi estimated that the user stayed near the starting point of the route. However, the proposed method followed the trajectory of the experimenter well. In general, the tracking accuracy of the Viterbi algorithm decreased with increasing MFI values, whereas the proposed method showed stable tracking results regardless of changes in MFI values. The average distance errors of two methods were 4.77 and 1.33 m, respectively.

The interesting thing about the tracking result images of the proposed method is that there was a gap between the predicted user locations. This is because, unlike Viterbi, the proposed method reflects the time intervals between fingerprints. More precisely, the proposed method considers the case where the data frame is not transmitted from the smartphone and predicts the location more accurately by selecting a location a little further away than simply a location next to it.

#### 5.2.2. Experiment on Route 2

**Figure 12:**
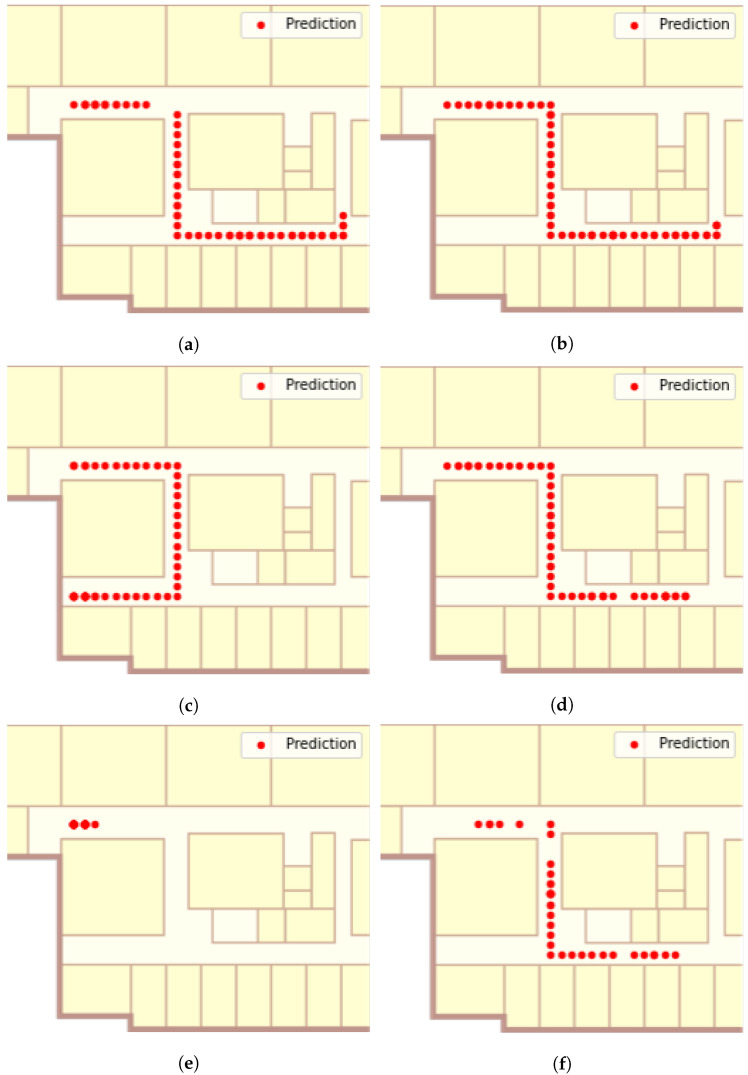
Tracking result on Route 2. (**a**) Viterbi to the packet generator, (**b**) proposed method to packet generator, (**c**) Viterbi to YouTube, (**d**) proposed method to YouTube, (**e**) Viterbi to Instagram, (**f**) the proposed method to Instagram.

In Route 2, the accuracy of the two algorithms showed a greater difference because the route length was longer. As shown in Figure 12, compared to the experiments in Route 1, the accuracy of Viterbi significantly decreased. The Viterbi algorithm did not properly track the user’s movements, except for the packet generator scenario. Fortunately, in the YouTube scenario, the first corner turn was correctly predicted, but in the second, it chose a different direction. However, in the Instagram scenarios, the algorithm determined that the user was staying in the starting location of the route. In contrast, the proposed adaptive method showed strong tracking results for all scenarios. The average tracking accuracy of the Viterbi algorithm and the proposed method for all scenarios was 4.02 and 1.26 m, as presented in Table 3.

**Figure 13:**
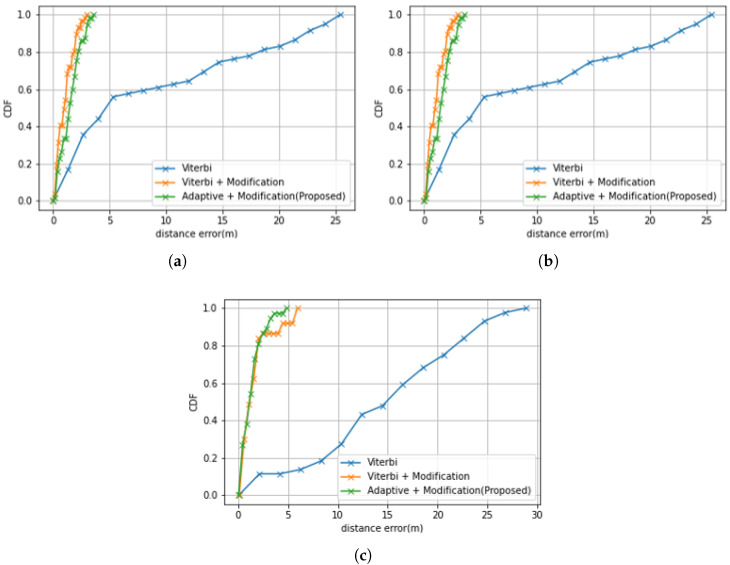
CDF of Route 2 experiments. (**a**) Packet generator, (**b**) YouTube, (**c**) Instagram.

Figure 13 shows the cumulative distribution function (CDF) for the distance error of all scenarios in Route 2. The three different graphs drawn on the CDF represent the tracking results of applying three different tracking methods: the Viterbi algorithm, Viterbi algorithm with the fingerprint modification process introduced in Section 4.4, and the proposed method, which utilized both the modification process and adaptive Viterbi. As shown in the figure, through the fingerprint modification process, the tracking error was quite reduced; except for the YouTube scenario, the proposed adaptive transition probability enhanced the tracking performance more.

## 6. Discussion

This paper proposes an adaptive user tracking method to solve an unstable tracking problem that occurs when a data frame is used for passive fingerprint positioning. Two main strategies were applied for the method, one is fingerprint modification and the other is the time interval adaptive tracking algorithm. Fingerprints generated with a fixed time window were filtered and supplemented through the proposed modification process. Subsequently, the trajectory of the user was inferred by applying the proposed tracking algorithm, which reflected the time difference between the adjacent modified fingerprints. The accuracy and stability of the proposed tracking method were verified through experiments on two different routes. In order to compare the tracking performance according to the time frame interval, four scenarios with different MFI values were set and the experiment was conducted. Viterbi, a popular tracking algorithm, showed moderate results only in the low MFI scenario while the frame proposed method perfectly ’followed up’ the locations of the user in all scenarios.

However, there were two limitations to this work. First, the accuracy of the proposed method depended on the speed of the target user. Although the proposed method provided partial robustness with parameters configured under the assumption of the maximum speed of 0.5 m/s, tracking results could be unstable at speeds much faster than it. Second, the target space for evaluation was relatively small for asserting practicality. When these future works are resolved, it is expected that people’s interests and preferences can be analyzed by identifying movements in indoor environments for more diverse and practical scenarios.

## Figures and Tables

**Table 1:** MFI of the experiments.

Route Type	Packet Generator	YouTube	Instagram
Route 1	1.54	2.42	3.41
Route 2	1.42	2.22	3.90

**Table 2:** Route 1 distance error.

Scenario Name (MFI)	Viterbi	Proposed Method
Packet generator (1.54)	1.01	1.04
YouTube (2.42)	4.56	1.35
Instagram (3.41)	8.75	1.59
Average	4.77	1.33

**Table 3:** Route 2 distance error.

Scenario Name (MFI)	Viterbi	Proposed Method
Packet generator (1.42)	1.95	1.15
YouTube (2.22)	8.19	1.94
Instagram (3.90)	14.45	1.28
Average	8.20	1.46

## Data Availability

Not applicable.
